# Vaccination intentions generate racial disparities in the societal persistence of COVID-19

**DOI:** 10.1038/s41598-021-99248-2

**Published:** 2021-10-07

**Authors:** Yanchao Wang, Alina Ristea, Mehrnaz Amiri, Dan Dooley, Sage Gibbons, Hannah Grabowski, J. Lee Hargraves, Nikola Kovacevic, Anthony Roman, Russell K. Schutt, Jianxi Gao, Qi Wang, Daniel T. O’Brien

**Affiliations:** 1 Department of Civil and Environmental Engineering, Northeastern University, Boston, MA 02120 USA; 2 School of Public Policy and Urban Affairs, Northeastern University, Boston, MA 02120 USA; 3 Boston Area Research Initiative, Northeastern University, Boston, MA 02120 USA; 4 Boston Public Health Commission, Boston, MA 02118 USA; 5 Center for Survey Research, University of Massachusetts Boston, Boston, MA 02125 USA; 6 Department of Sociology, University of Massachusetts Boston, Boston, MA 02125 USA; 7 Beth Israel Deaconess Medical Center, Boston, MA 02215 USA; 8 Department of Computer Science, Rensselaer Polytechnic Institute, Troy, NY 12180 USA

**Keywords:** Health policy, Public health, Quality of life, Risk factors

## Abstract

We combined survey, mobility, and infections data in greater Boston, MA to simulate the effects of racial disparities in the inclination to become vaccinated on continued infection rates and the attainment of herd immunity. The simulation projected marked inequities, with communities of color experiencing infection rates 3 times higher than predominantly White communities and reaching herd immunity 45 days later on average. Persuasion of individuals uncertain about vaccination was crucial to preventing the worst inequities but could only narrow them so far because 1/5th of Black and Latinx individuals said that they would never vaccinate. The results point to a need for well-crafted, compassionate messaging that reaches out to those most resistant to the vaccine.

Ever since the World Health Organization declared COVID-19 to be a global pandemic on 11 March 2020, vaccination has been the light at the end of the tunnel. Following an unprecedented development effort by the biotech industry, the first vaccines became available in some countries for health-care workers and the most at-risk in winter 2020–2021. The United States, United Kingdom, and many other nations began to distribute vaccines to high-risk groups shortly thereafter and then extended distribution to the general public in the spring. This process is intended to curtail the rate of infection and is hoped to eventually eliminate infections by creating herd immunity. But an available vaccine does not automatically create herd immunity. If a sufficient fraction of the population does not choose to be vaccinated^1, 2^, substantial numbers of people will remain susceptible and infections will persist.

A series of surveys in late 2020 revealed that ~ 30–40% of Americans were hesitant to get vaccinated against COVID-19^3–5^; this mirrors results in other countries^6–10^. Some respondents were worried about receiving a vaccine before seeing more widespread evidence that it is effective and has few side-effects^5, 7, 9^. For example, a December 2020 KFF COVID-19 Vaccine Monitor survey reported that among the 27% of the U.S. population likely to not get the vaccine, 59% were worried about side effects and 55% did not trust the government to make sure that the vaccine is safe and effective^11^. Others stated that they are opposed to being vaccinated altogether, following a growing trend in American society to reject vaccines as having dangerous side effects^12, 13^. Resistance to COVID-19 vaccination is particularly pronounced among Black Americans, who cite a long history of discrimination and mistreatment at the hands of medical professionals^14, 15^.

The various objections to vaccination and their uneven distribution across communities have posed a major challenge for the pursuit of vaccine-based herd immunity. Of particular concern, differences between communities in willingness to be vaccinated will multiply the risks faced by residents and reduce the chances of achieving herd immunity locally for all. Specifically, if too few Black and Latinx individuals choose to be vaccinated, communities with many Black and Latinx residents will remain vulnerable to infection at disproportionate rates while communities that are predominantly White and Asian approach herd immunity. Such a disparate result would be yet another racial inequity in a pandemic that has already had a glaringly disparate impact on health and finances in Black and Latinx communities^16–20^. Further, communities are not islands unto themselves, and intermixing through the daily movements of individuals have been critical in explaining infection transmission across regions^21–26^. As such, if *any* community in a region fails to achieve a sufficient level of vaccination, it could harbor infections that still pose a risk to unvaccinated individuals in other communities through mobility-based transmission.

The current study evaluates the inequities that might arise from differential willingness to be vaccinated across a single metro region. We use a traditional SIR (susceptibility–infection–recovery) model for simulating the evolution of an infectious disease within a community, applied across the ZIP codes of Boston, MA, and the surrounding municipalities in the greater Boston area (collectively referred to as “communities” from hereon). We further inform these models using mobility data generated by cell phones to track how movement between communities could further spread the disease^21–26^. We incorporate into these models an additional set of parameters for the gradual rollout of a vaccine, which removes individuals from a community’s susceptible population at an assumed rate of effectiveness, based on clinical trials^27^. We simulate the vaccination rollout as if it had occurred October-December, 2020. This matches the proposed 3-month vaccination process for the general population that many leaders promised in early 2021 and allows us to leverage historical mobility and infections data and, as has been demonstrated by other simulation studies of vaccination^28^, permits us a clear counterfactual against which we can compare the introduction of vaccination.

The study is designed to address four main research questions, each of which is relevant to the initial vaccination rollout for COVID but can also be generalized to future vaccination rollouts for this or other pandemics. First, we simulate both the global and community-specific rollout of vaccines, revealing at what point the process would be expected to hit a “bottleneck,” where supply outstrips willing recipients, and whether this milestone arrives at different times across communities. This will be crucial to leaders seeking to manage vaccine supply. Second, we quantify the anticipated impact of racial differences in vaccination hesitancy, which are largely attributable to historical inequities in medical treatment. To do so we use results from three recent surveys that separated responses by race to approximate willingness to vaccinate in each community. Third, there is evidence that those who are uncertain about receiving a new medical procedure, including vaccinations, often wait until others that they know have done so with few negative side effects^29–31^. In order to examine both the impact and limitations of this process, we include a “persuasion” rate (or “imitation” factor^32, 33^) by which individuals who were uncertain if they would get vaccinated can eventually decide to do so as the proportion of those vaccinated in their community increases. Fourth, a major concern is that slow uptake of vaccination in one community can undermine herd immunity in neighboring areas via mobility-based exposure. Here we will be able to evaluate this proposition.

## Results

### Vaccination

**Figure 1:**
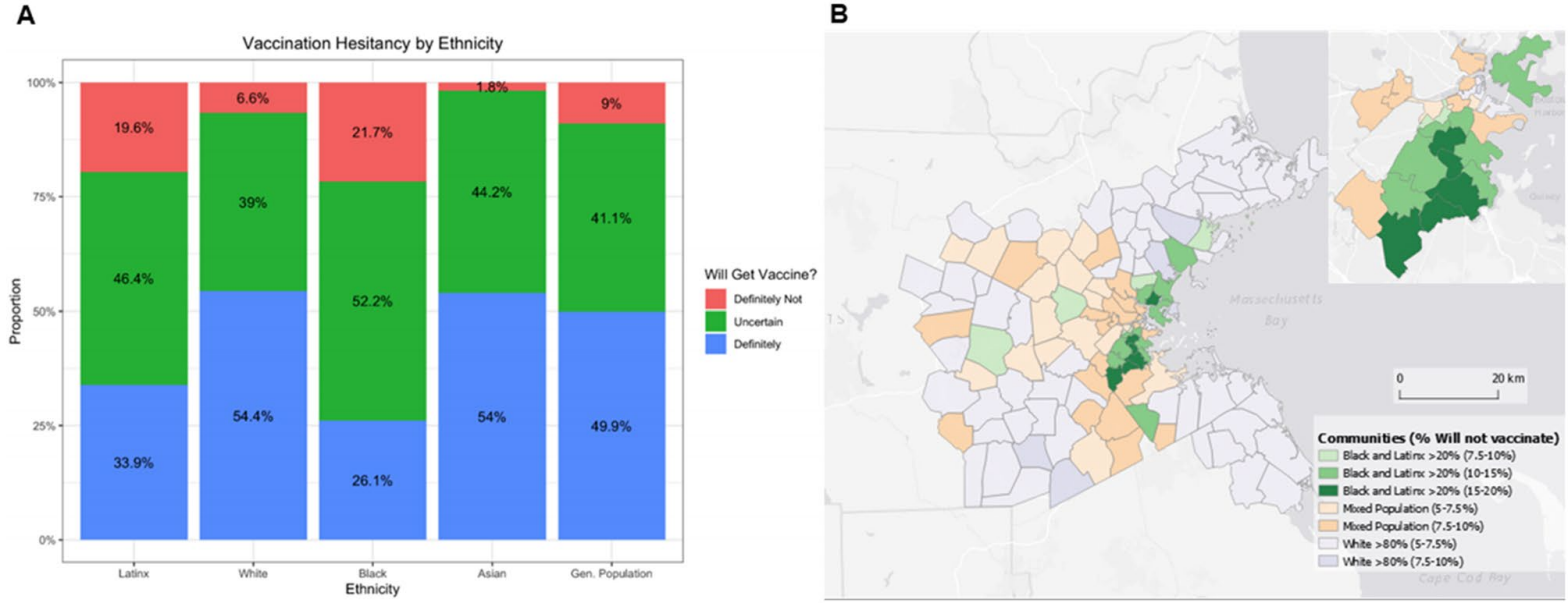
Representation of variations in vaccine intentions. (**A**) Individuals vary in their intentions to receive the vaccine by ethnicity. When combined with (**B**) the categorization of communities by predominantly White (> 80%), high Black–Latinx (> 20%), or other, these disparities translate into geographic differences in the proportion who definitely will not vaccinate. (Made in R Studio V.1.4. http://www.rstudio.com/).

In three independent surveys of Boston and Massachusetts residents in the fall of 2020, 49.6% of respondents said that they planned to get the COVID-19 vaccine, and 8.8% said that they did not; the remaining 41.6% were uncertain. These responses featured prominent disparities by race, however. On the low end, 6.6% of White respondents and 1.8% of Asian respondents said they would definitely not get the vaccine, compared to 21.7% of Black and 20% of Latinx respondents (see Fig. 1A). We combined these ratios with the racial composition of communities to estimate the percentage of residents in each who planned to get vaccinated, did not plan to, and who were uncertain. This revealed stark differences across communities, with municipalities in the region varying between 33.9 and 54.1% of the population saying they would definitely get vaccinated, and between 5.4 and 17.2% saying they definitely would not; the same ranges indicated slightly less receptivity of vaccines in Boston’s ZIP codes, which has a higher Black and Latinx population than most surrounding municipalities (definitely will: 28.2–52.1%; definitely will not: 5.1–19.8%; sees Fig. 1B, Figure S1a–c).

We simulated the impact of vaccination across communities by incorporating it into an SIR-mobility model with parameters approximating transmission and recovery rates based on actual case numbers for October-December 2020. We also assume the 95% efficacy of the vaccine reported by the initial clinical trials^27^ and that vaccination will occur at the rate of 8.25% of the population per week, following the goal of complete vaccination over a 12-week (3-month) period. The model also allowed for those uncertain about the vaccine to be persuaded, contingent on the likelihood of seeing others in their neighborhood who had been vaccinated and presumably not seen adverse side effects^31^. To maintain a true counterfactual, all comparisons of outcomes are made against the results of the same model without vaccination as a facsimile for the actual events of this time period.

**Figure 2:**
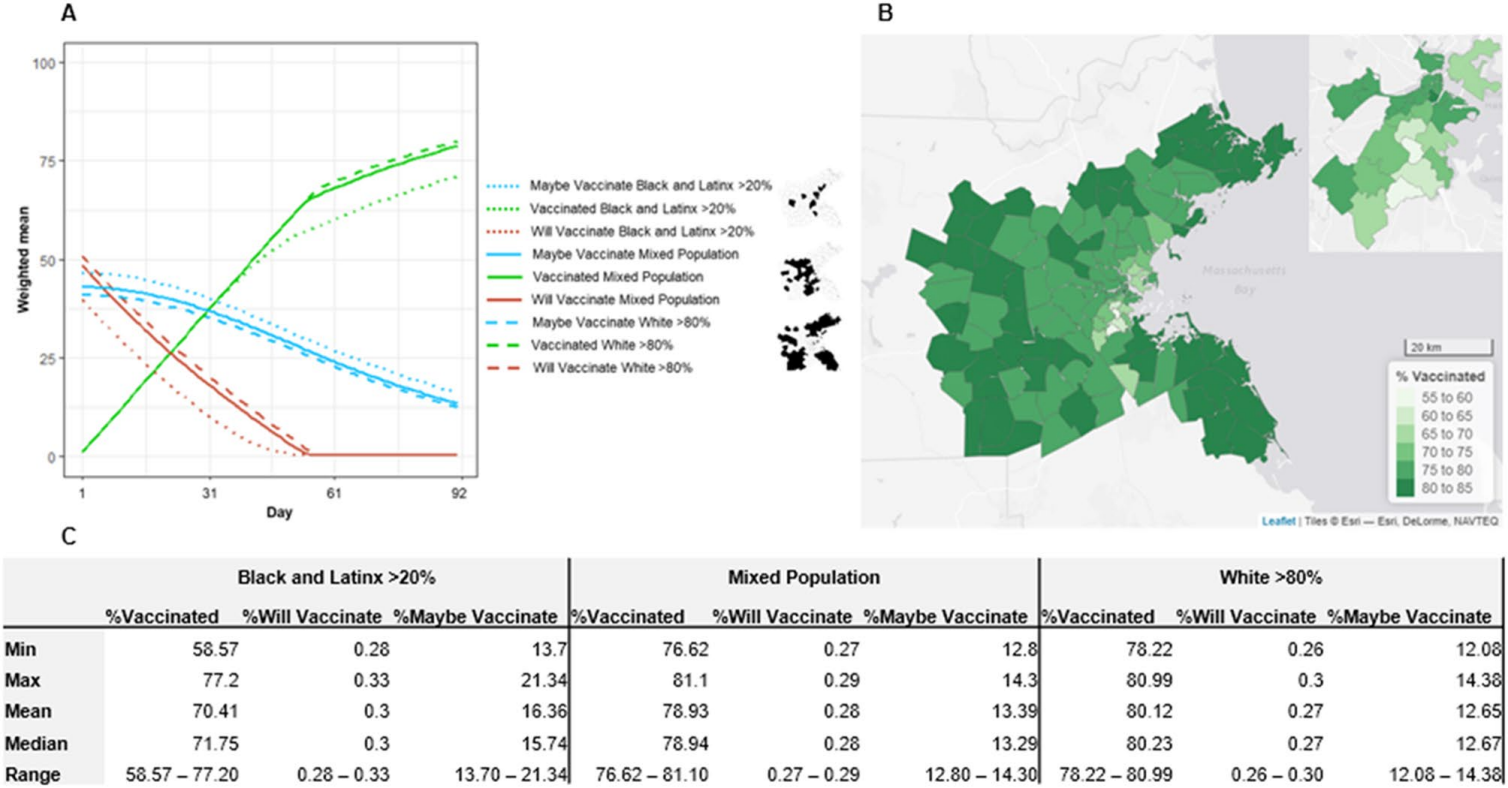
Vaccination rates varied across communities throughout the simulation. (**A**) Growth curves of the percentage of residents intending to vaccinate, will maybe vaccinate, and have been vaccinated across the 3-month simulation, broken out by communities that are predominantly White, high Black–Latinx, and other. These different curves resulted in disparities across communities in (**B**) the total percentage of those vaccinated at the end of the simulation. (**C**) Shows the disparities at the end of the modeling process. (Made in R Studio V.1.4. http://www.rstudio.com/).

Figure 2A depicts a steady increase over the 3-month period in the proportion of people who were vaccinated across the region, reaching 75%. In late November, however, the vaccination process hit a bottleneck (*mean* = 53 days into simulation). It had exhausted all individuals who either were willing to be vaccinated at the outset or were persuaded to that point, as indicated by the blue line reaching zero. As a result, vaccination from then on was dependent on those additional individuals who were persuaded in each week, which was less than the rate at which vaccination was possible. This created the kink in the red line, indicating a slowed vaccination process from that point on, explaining why the simulation did not successfully vaccinate 100% of the population after 12 weeks, despite having the capacity to do so.

The bottleneck in vaccination did not occur at the same time in all communities (see Fig. 2B,C). For purpose of comparison here and moving forward, we divide communities into those that are predominantly White (> 80% White residents; 51% of communities), those with high Black–Latinx populations (> 20% Black and Latinx residents; 17% of communities), and those that are neither (32% of communities). For predominantly White communities, the bottleneck was reached at the very end of November (*mean* = 57th day). Meanwhile, in communities with high Black–Latinx populations the same milestone occurred before November 15th (*mean* = 42nd day). Because this date was reached earlier, the lower proportion of vaccinated residents at that time in turn resulted in a diminished power of persuasion, meaning fewer additional people were persuaded each week thereafter than in predominantly White communities. This further exacerbated disparities in cumulative vaccinations. By the end of the simulation, residents in predominantly White communities were consistently 80% vaccinated whereas those living in high Black–Latinx populations were 71% vaccinated, though there were communities with rates of vaccination as low as 59%.

### Infection rates

**Figure 3:**
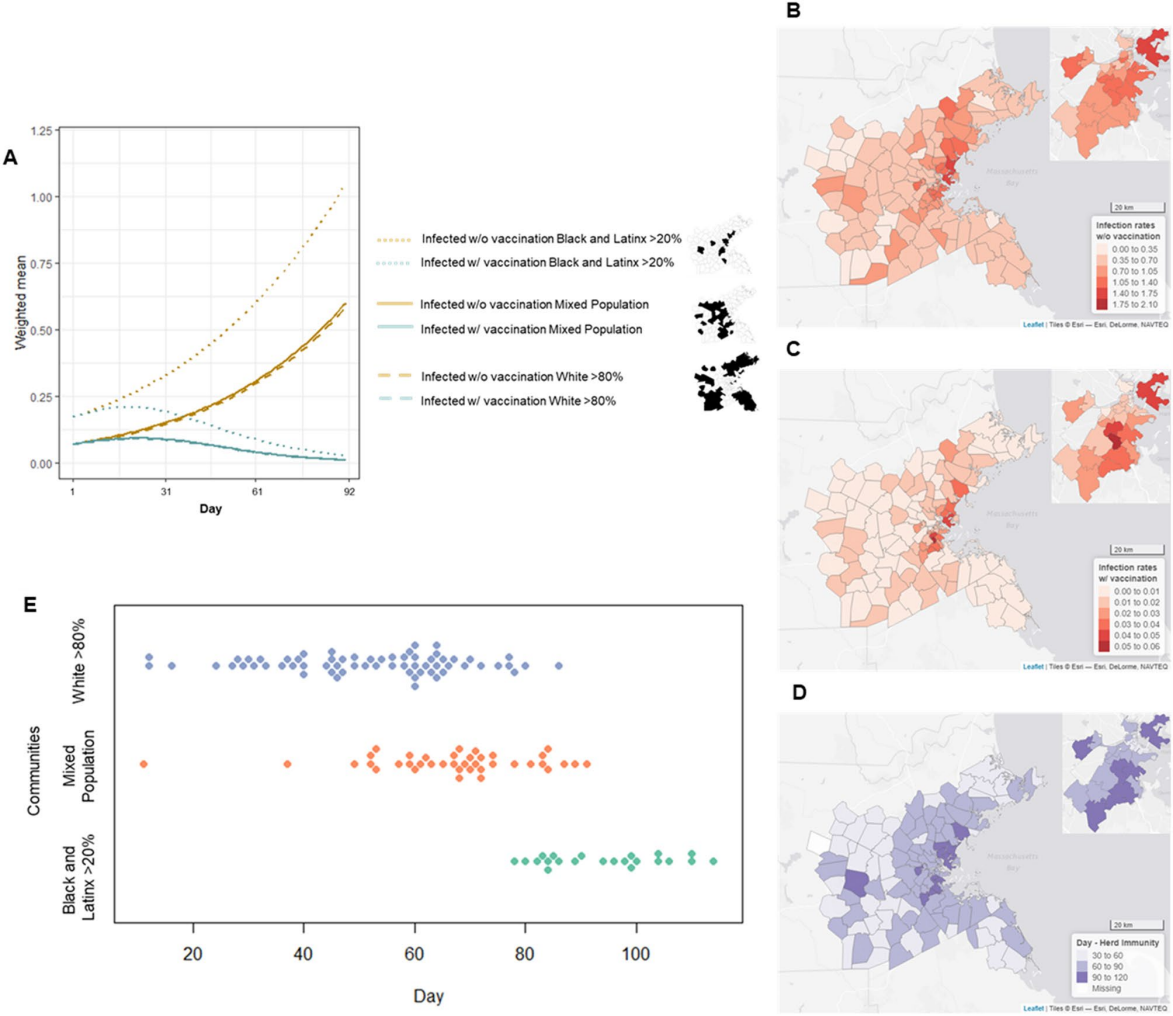
Vaccination rates varied across communities throughout the simulation. (**A**) Growth curves of the percentage of residents intending to vaccinate, will maybe vaccinate, and have been vaccinated across the 3-month simulation, broken out by communities that are predominantly White, high Black–Latinx, and other. These different curves resulted in disparities across communities in (**B**) the total percentage of those vaccinated at the end of the simulation, (**C**) infection rates at the end of the modeling process, (**D**) and the day when herd immunity was reached, while (**E**) shows a graphic representation of when communities reached herd immunity. (Made in R Studio V.1.4. http://www.rstudio.com/).

The impacts of vaccination, including disparities in uptake across communities, were evident in the corresponding evolution of infection rates. As shown in Fig. 3A, infection rates kept pace with the no-vaccination scenario until mid-October, which is when vaccination began to substantially lower the population susceptible to infection. Over the following week or so, infection rates started to decrease, eventually nearing zero infections. To wit, 99% of communities had more than one case per 100 without vaccination, whereas no community was above 0.75 cases per 100 with vaccination.

The growth curve for infection rates under vaccination was consistent across communities of different racial composition, though with some noteworthy variations. First, the absolute drop in infection rates with vaccination was greater in communities of color, seemingly because they had much higher levels of infection in the no-vaccine scenario. In other words, vaccination held the greatest absolute benefit for communities harder hit by the virus. The second difference, however, was how close they came to herd immunity. Predominantly White communities nearly reached zero infections (3rd quartile = 0.10 cases per 100 residents). Meanwhile, communities with high Black–Latinx populations failed to reach this point, averaging 0.27 infections per 100 residents at the end of the simulation (Fig. 3A–C).

A series of linear regression models confirmed the racial disparities in infection rates under the vaccination scenario (see Table 1 for all parameters). Communities with greater Black and Latinx populations had higher infection rates at the conclusion of the simulation (% Black: B = 0.30, *p* < .001; % Latinx: B = 0.54, *p* < .001). Even when we controlled for expected infection rates, as drawn from the no-vaccine scenario, these racial disparities persist, meaning that differential uptake of the vaccine played a consequential role in cross-community variations in infection (% Black: B = 0.26, *p* < .001; % Latinx: B = 0.26, *p* < .001).

**Table 1:** Parameter estimates from regression equations using demographic composition, mobility-based exposure, and expectations from the no-vaccine scenario to predict infection rates at the end of the vaccination simulation.

	Unstand. beta (S.E.)	β	Unstand. beta (S.E.)	β	Unstand. beta (S.E.)	β	Unstand. beta (S.E.)	β
Exp. infections^a^	–	–	0.02*** (0.001)	0.53	–	–	0.02*** (0.001)	0.61
% Black	0.30*** (0.03)	0.42	0.26*** (0.01)	0.37	0.26*** (0.03)	0.36	0.28*** (0.01)	0.39
% Latinx	0.54*** (0.04)	0.63	0.26*** (0.02)	0.30	0.50*** (0.04)	0.58	0.23*** (0.02)	0.27
% Asian	− 0.05 (0.04)	− 0.04	− 0.07*** (0.02)	− 0.06	− 0.10* (0.04)	− 0.09	− 0.05* (0.02)	− 0.04
Mobility-based exposure^b^	–	–	–	–	0.02*** (0.004)	0.22	− 0.01*** (0.002)	− 0.10

For 128 communities, defined as the municipalities of the greater Boston region and the ZIP codes within Boston.

*p < .05; ***p < .001.

^a^Based on rates in the no-vaccine scenario.

^b^Potential exposure to infection transmission via movement between communities, as derived from the model parameters.

### Achieving herd immunity

Moving beyond infection rates, did communities reach the goal of herd immunity via vaccination? We defined herd immunity as the point at which a community effectively eliminated the virus locally (i.e., < 1 infection; the model permits fractions of infections). We found that only 27% of communities had reached herd immunity by this definition at the end of the simulation. We thus extended the simulation for three additional months (simulating mobility based on historical data; see Methods for more). An additional 52% of communities achieved herd immunity in the fourth month of the extended simulation (80% cumulative), and all but one remaining community achieved herd immunity in the fifth month, which reached it shortly thereafter (see Fig. 3D,E). The average community reached herd immunity on day 103 of the simulation.

Differences in achieving herd immunity again reflected stark disparities by race. Only 14% of communities with high Black–Latinx populations saw herd immunity before the fifth month of the simulation, whereas 99% of predominantly White communities had achieved herd immunity by this time. To reiterate, *all* predominantly White communities achieved herd immunity before *any* community with a high Black–Latinx population, some by nearly 2 months. The average difference in achieving herd immunity between these two sets of communities was 45 days (89 days and 134 days into the simulation).

### Mobility-based exposure

Mobility-based exposure—that is, the quantification of a community’s potential exposure to infection via cross-community travel—has been a central component of models of the transmission of COVID^21–26^ and we included it in our own. Unsurprisingly, when added to the previous regressions, mobility-based exposure in a community at the end of the simulation independently predicted higher infection rates, albeit with an effect size considerably lower than those of racial composition (β = 0.22, *p* < .001; see Table 1 for all parameters). The inclusion of mobility-based exposure in the model did not meaningfully alter the effects of race. However, when we controlled for infection rates in the no-vaccine scenario, mobility-based exposure had a *negative* effect on infection rates under vaccination (β = − 0.10, *p* < .001). This would appear to be because vaccination especially benefited those who were more at risk from either baseline or dynamic exposure. This would all indicate that mobility-based exposure had a moderate effect on continued infections, though it played a much smaller role relative to vaccination in determining infection trends. As a final test of this interpretation, we ran a model using only percentage vaccinated and mobility-based exposure to explain infection rates, and found that the effect of the former was four times the size of the effect of the latter (β = − 0.78 vs. β = 0.21, both *p* values < .001).

If mobility-based exposure and vaccination are each relevant to the evolution of infections, the question remains how often these two factors coincide, making certain communities doubly vulnerable. We find that mobility-based exposure correlated moderately with the proportion of Black and Latinx residents (% Black: *r* =0. 23, *p* < 0.01; % Latinx: B = 0.23, *p* <0.01) and was slightly negatively associated with vaccination rates (*r* = −  0.28, *p* < 0.01). Thus, although high levels of mobility-based exposure and vaccination do not always coincide, the communities where they do are at highest risk.

### Robustness tests and implications

In order to probe the robustness of the simulation, we tested higher and lower levels of persuasion, as well as no persuasion; two lower levels of vaccine efficacy; and two longer timelines for vaccination roll-out. We re-ran the models with all possible combinations, making for 36 sets of results (4 × 3 × 3). These alterations had the expected effects on global patterns, either extending or compressing vaccination time, or increasing or decreasing total infections. The disparities in infection rates and herd immunity were also present across models. We conducted a meta-analysis of the disparities in infection rates and the attainment of herd immunity, entering the levels of persuasion, vaccine efficacy, and distribution time into regressions predicting specific outcomes and parameters: the effects of % Black and % Latinx on infection rates, both with and without controlling for infection rates under the no-vaccine scenario; the difference in the proportion of predominantly White and high Black–Latinx communities that reached herd immunity; the difference between predominantly White and high Black–Latinx populations in the average date on which herd immunity was reached.

**Figure 4:**
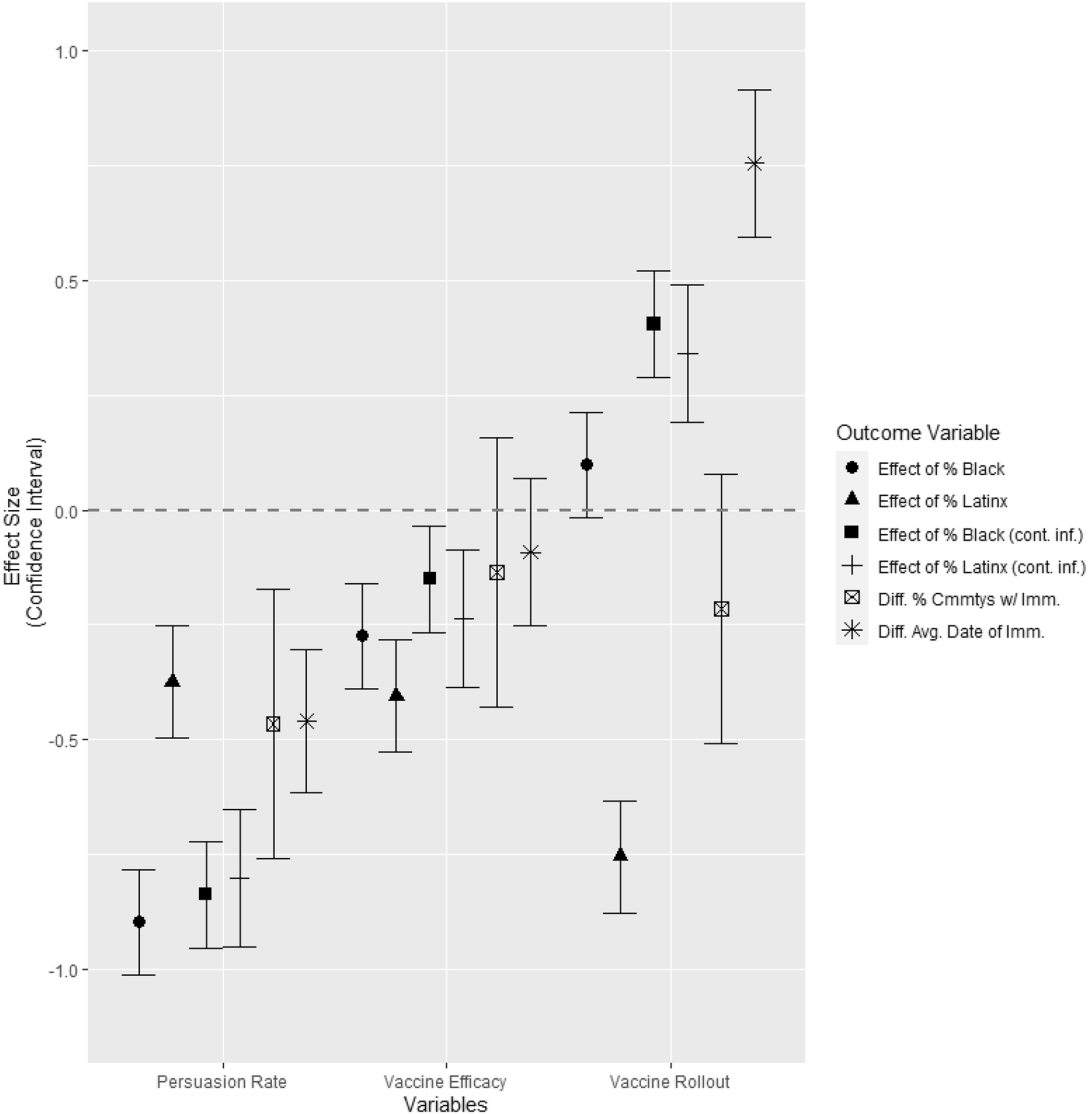
Disparity across simulations. Parameter estimates (unstandardized betas, standard errors, and standardized betas) from regression equations meta-analyzing the indicators of disparity from across simulations with different levels of persuasion, vaccine efficacy, and rollout rate.

As illustrated in Fig. 4, the level of persuasion had the strongest effect, markedly lowering all measures of disparity (all *p* values < .0.001). Greater vaccine efficacy also lowered the association between percentage Black and Latinx population and infection rates, both in general and owing uniquely to the introduction of vaccination itself (i.e., relative to the no-vaccine scenario). Vaccine efficacy had little effect on disparities in reaching herd immunity, however. This was likely because less effective vaccines will extend the timeline to herd immunity for everyone, but the burden of allowing infections to persist will fall heaviest on those communities experiencing the most exposure. Last, a quicker rollout rate led to *greater* disparities when controlling for infections in the no-vaccination scenario and differences in the average date of reaching herd immunity. This is likely because high Black–Latinx communities reach the vaccination bottleneck sooner and thus fall behind their predominantly White counterparts at a faster rate. It is important to note, however, that the absolute outcomes were better for everyone under this scenario. Last, a faster rollout led to lower disparities in the raw infection rate for the Latinx population, but this appeared to attributable to the higher starting infection rate and mobility-based exposure in these communities, making a quick rollout especially valuable.

As we describe these differences across simulations, we must take into account their practical implications. The strength of persuasion is the most important component in the models, and it cuts both ways. If we remove persuasion entirely, 97% of predominantly White communities reach herd immunity in the 180-day simulation, with an average of 121 days, whereas no high Black–Latinx communities do. However, its power to eliminate disparities seems limited. When we increased the rate of persuasion by 50% from the baseline model, the average high Black–Latinx community saw herd immunity only 9 days earlier, while predominantly White communities saw an even smaller improvement of 3 days. This closed the gap between the two by only 14%. This indicates that even if individuals uncertain about getting vaccinated are convinced to do so more quickly, the proportion of people committed to being vaccinated at the outset and, conversely, the proportion committed to not being vaccinated, are far more consequential in determining the timeline of reaching herd immunity.

## Discussion

The simulation found that the introduction of vaccinations helped all communities. Disparities, however, between predominantly White communities and communities of color emerged early and worsened as the simulations proceeded, the details of which answered each of our four research questions. First, communities quickly reached a bottleneck in vaccination, at which point further vaccination relied entirely on continued persuasion. Because communities with more Black and Latinx residents had fewer people who were initially willing to be vaccinated, they reached this bottleneck weeks before predominantly White communities. Second, the disparities in vaccination rates set the stage for inequities in infection rates. Communities of color experienced average rates of infection 3 times as high as predominantly White communities and reached herd immunity on average a month-and-a-half later. Third, we found that persuasion is crucial to closing the infection gaps between communities of different racial backgrounds. Unfortunately, however, increasing the power of persuasion had limited additional effect because of the large number of people saying they would definitely not get vaccinated in communities of color. Fourth, mobility-based exposure had only a moderate impact during vaccination, meaning that lower adoption of vaccines in some communities did not heavily undermine herd immunity in surrounding areas.

We proceed by elaborating on the insights from each research question in greater detail, but we must note that much has happened since the beginning of the vaccination program and the original execution of this study. Vaccination programs have been successfully administered nationwide, verifying much of what the simulations predicted. We conducted a posthoc analysis using data published by the Massachusetts Department of Public Health (weekly data available at https://www.mass.gov/info-details/massachusetts-covid-19-vaccination-data-and-updates#weekly-covid-19-municipality-vaccination-data-) and found that our estimated rates of vaccination by community correlated substantially with those reported on August 26, 2021 (*r* = .0.53). This is noteworthy because our analysis relied solely on race and ethnicity. In fact, the simulation results mapped almost perfectly onto correlations between actual vaccination rates and the racial composition of communities, the only deviation being that we *underestimated* the willingness (or willingness to be persuaded) to be vaccinated among Asian and Asian American residents (correlations of indicators of ethnic composition with residual variance in actual vaccination rates not predicted by our simulations: % Asian: *r* = 0.24, *p* <0.01; % Black: *r* = − 0.02, *p* = *ns*; % Latinx: *r* =0.06, *p* = *ns*; % White: *r* = − 0.10, *p* = *ns*.). In addition, bottlenecks in vaccination occurred as the population of willing recipients dwindled in the late spring; infections have persisted in areas with lower vaccination levels; and persuasion did appear to wane as those who said they would never get vaccinated remained steadfast. Political resistance to vaccination among conservative White populations has also emerged as a major barrier to herd immunity that we did not consider in our projections (though it may not have had much impact with our data given the generally liberal politics of the Greater Boston area). That all said, the emergence of new variants, especially the now-predominant, and substantially more virulent and contagious Delta variant, has altered the landscape of the battle against COVID-19. Thus, we conclude by discussing implications for the current moment, which largely exacerbate nuanced emergence and persistence of inequities that we forecast here.

Turning to our first research question, the bottleneck in vaccination raises two points worth elaborating. From a logistical perspective, the assumptions of our model were such that persuasion could not keep pace with vaccine supply, creating implications for proper management of distribution. Meanwhile, from a social perspective, the earlier bottleneck in communities with more Black and Latinx residents meant that they had fewer people vaccinated at this point. Because the persuasion rate is dependent on the proportion of community members who have been vaccinated, these communities fell further and further behind in vaccination in the following weeks. Thus, for both reasons of supply management and long-term advancement of the rollout, the bottleneck is a key moment in the evolution of the model and policymakers and practitioners should be attuned to any signals that this point is arriving and to ramp up community messaging accordingly. This issue was observed regularly in the early months of the vaccination rollout and has resulted in millions of wasted doses^34^. It could easily become an issue again if and when society moves to re-vaccinate owing to waning immunity or vaccines that are more efficacious against variants.

Because of their lower levels of vaccination in the simulations, communities of color were faced with a greater and more persistent threat of the virus than predominantly White communities. It is important to note the full meaning of this statement. All communities saw heavily diminished infection rates over the 3-month simulation and eventual herd immunity by the end of 6 months. But it took communities of color longer to arrive at that point, meaning they experienced a substantially greater cumulative amount of infections (and, consequently, deaths) than predominantly White communities. Thus, this is neither a doomsday scenario, nor is it what any mayor or governor would want for their city or region.

With that in mind, persuasion will be most critical to planning and leadership moving forward. By varying the strength of persuasion across models we see just how important it is to outcomes in the vaccination rollout^30, 31, 33^. The starkest illustration of this is that when we removed persuasion from the model, no high Black–Latinx community reached herd immunity by the end of 6 months, compared with over 95% of predominantly White communities. More subtle, but equally telling, was the impact of increasing the rate of persuasion as it only marginally narrowed inequities. This is because our model reflected a leading strategy of focusing on those who are unsure about getting the vaccine. This strategy operates on the assumption that those who say they “definitely will not” get the vaccine cannot be convinced by any quantity of evidence^10, 35^. Given the proportion of people who have said (and continue to say, at the time of the final revision) that they will never get vaccinated, especially in communities of color, this makes for a precarious situation; this is also increasingly a concern in more conservative white communities.

If one follows the logic of taking those that say they will never get vaccinated at their original word for Boston, 1 in 5 Black and Latinx residents would never be vaccinated. This would pose a large stumbling block to achieving herd immunity for communities of color. Though we have estimated herd immunity as the first moment at which there are no infections in a community, there could still be pockets of low vaccination with concomitant gaps in the protective wall of community health^36^*.* That said, vaccine hesitancy is not synonymous with vaccine denial, which questions efficacy and propagates potential harms from vaccines^37^. With luck, some of those who are rejecting the idea of being vaccinated now are more hesitant than they are deniers, and they will be receptive to increased evidence of the vaccine’s effectiveness and safety. Thus, the solution is not only to increase the rate at which people on the fence decide to be vaccinated; it is in convincing those steadfastly opposed to the vaccination to consider it. This has only become more critical since vaccine rollout began as misinformation has steadily undermined efforts to reassure those who are hesitant^38^.

Our fourth research question regarded the implications of lower vaccine adoption in some communities for the surrounding region. Namely, could lower adoption in one or more communities undermine the pursuit of herd immunity in surrounding communities? This would seem plausible given the known importance of cross-community mobility for transmission of the virus^21–26^. We find here a mixed answer. Indeed, mobility-based exposure continued to predict infection rates, though with a strength far smaller than that of vaccination adoption. This is consistent with other work that has found that the importance of mobility has been largely supplanted by the impact of interventions intended to mitigate transmission of the virus, whether they be social distancing guidelines in the summer of 2020 or, in this case, vaccine rollouts^39^. As such, the ability of a community with lower vaccine adoption to impact other communities around it appears to have been limited under the assumptions of the model (though see below for more on the implications of the Delta variant).

There are a number of limitations to the model and its implications arising from assumptions we were forced to make. First, we did not account for any first-stage rollout to first responders, frontline workers, and the most at-risk, which was anticipated to reach ~ 10–15% of the population. This would accelerate herd immunity by raising the baseline level of vaccination and kick-starting persuasion. Second, we assumed that vaccinated individuals cannot carry the virus. When this study was originally conducted, there was little evidence one way or the other as to whether this was the case, though this has now changed with the emergence of the Delta variant. Third, our simplistic model of persuasion ignores the power of leadership, which is argued to be as critical in communities of color as seeing one’s neighbors be vaccinated^40^. Fourth, the reproduction rate of the virus remained stable throughout the simulation, but there are reasons to believe that vaccination leads to behavioral shifts that will increase the underlying reproduction rate even if vaccines themselves limit the total impact^41^. Our estimates of infection rates and the timeline to herd immunity could be somewhat optimistic in that case. Fifth, the model treats vaccination access as being evenly distributed across communities, whereas numerous analyses have found lower numbers of vaccination sites in communities of color in multiple U.S. cities, especially in southern states^42, 43^. All of these dynamics could alter the specific quantitative results we see here and the timeline to herd immunity. That said, given the variety of robustness tests that we have run, we are confident saying that in any of these scenarios there will be extensive inequities in infection rates and the time elapsed before achieving herd immunity between predominantly White and Asian communities and Black and Latinx communities—and that some of these nuances would only exacerbate outcomes.

We conclude by noting the new shape the pandemic has taken in the months since the vaccination program started and this study was originally conducted. As described above, our simulation forecast inequitable outcomes, but eventual herd immunity for everyone. Unfortunately, since the emergence of the more virulent Delta variant, this situation no longer seems near at hand, if even within the realm of possibility. The variant is far more virulent, instigating what the governor of Mississippi called “a pandemic of the unvaccinated,” but it is also more contagious, lowering the efficacy of vaccination. We did run alternate simulations that iteratively lowered levels of vaccine efficacy, and these increased racial inequities in the long-term persistence of infections. Additionally, lowering vaccine efficacy to 75% increased the impact of mobility on infection rates by ~ 25%. Greater virulence would only magnify each of these findings, further highlighting the urgency around inequities but also tempering our forecast that inter-community mobility would be less of a concern at this point in the vaccination program.

Last, the narratives surrounding vaccination intentions in the United States have shifted somewhat, with increasing concern not only about hesitance among Black and Latinx residents in urban areas, but also among conservative White Americans, especially in rural areas^44^. Simulations like those run here that address whatever local disparities exist could be useful for planning across the United States and internationally, provided that there are tools available for tracking intentions, be they repeat surveys (especially with panel designs^45^), social media polls^46^, or otherwise. This is especially true if there is a transition to the distribution of booster shots and widespread revaccination, in which case attitudes may be somewhat different from before. In any case, the pandemic has not in fact been “ended” by the current vaccination program and continuing to refine these multi-methodological approaches to anticipate and respond to it will be crucial.

## Materials and methods

The study centers on an SIR model that uses pre-determined transmission and recovery rates in conjunction with mobility between communities to estimate daily infection rates in each community. The models were run for October-December, 2020, and the transmission and recovery rates were based on actual infection records. Mobility was also derived from historical data for the same time period. The “communities” act as nodes in the mobility model and are defined as the 100 non-Boston cities in towns in the greater Boston region (following the Metropolitan Area Planning Council’s definition) and the 28 ZIP codes within Boston. This decision was made based on the availability of more granular data for Boston as well as the large amount of between-ZIP code demographic diversity within the city, which is lower or absent in many of the surrounding municipalities.

### Data and measures

The models used four data sources: (1) population descriptors from the American Community Survey’s 2014–2018 5-year estimates; (2) daily and weekly infection case counts, derived from infection records, for all towns in greater Boston and ZIP codes within Boston; (3) responses to three surveys including items on people’s intentions regarding vaccination; (4) cross-community mobility records derived from cell phone records, generated by SafeGraph, a data company that aggregates anonymized location data from numerous applications in order to provide insights about physical places, via the Placekey Community. To enhance privacy, SafeGraph excludes census block group information if fewer than five devices visited an establishment in a month from a given census block group. The Boston Area Research Initiative’s Geographical Infrastructure^47^ was used to join data describing each municipality or ZIP code.

#### Census indicators

We drew population descriptors from the U.S. Census’ American Community Survey’s 2014–2018 estimates for all census block groups in Massachusetts. This level was selected as it is the largest census geography that nests cleanly within ZIP codes in Boston and within municipal boundaries. Community indicators included total population and ethnic composition (i.e., proportion Asian, proportion Black, proportion Latinx, proportion White). All measures were aggregated from census block groups to the municipal or ZIP code level using population-weighted means.

#### Infection cases

The Commonwealth of Massachusetts’ Department of Health released weekly counts of new infections for all municipalities, starting on April 14th, 2020. It also released daily counts for counties. From these two data sources we created daily town measures by: tabulating the weekly sum of infected cases in a county; calculating the percentage of a county’s cases attributed to each town; estimating the daily infected cases per town as the same percentage of the daily count for the county. For Boston ZIP codes, we had case records tracked by the Boston Public Health Commission mapped to the ZIP code of residence. We tabulated these for daily counts.

#### Surveys

Three surveys of Massachusetts residents were conducted that included a question regarding intention to vaccinate and split these responses by race. The three surveys were conducted by the Center for Survey Research at University of Massachusetts Boston with the Boston Area Research Initiative, MassInc Polling on behalf of the Boston Museum of Science, and Suffolk University Polling and the Boston Globe. The first surveyed residents of Boston from September to November and the other two surveyed residents from throughout Massachusetts in November and December, respectively. Although the exact wording varied between them, all three surveys permitted respondents to say that they “definitely” or “definitely did not” plan to get the vaccine or that they were uncertain or undecided. The overall proportion of individuals falling in each of these three groups was consistent across the three surveys, as were the breakdowns by race (though the MassInc poll did not have enough Asian respondents to include cross-tabs for that group; see Supplementary Online Materials for full results).

We summed the cross-tabs for the vaccination intention question by race across the three surveys to calculate the weighted proportion of individuals indicating “as soon as possible,” “never,” and something in between for each of the four major racial categories—Asian, Black, Latinx, and White. We then estimated the proportion of residents in each of these three categories regarding the vaccine for each municipality and Boston ZIP code with the following equation:

$$Y_{i,k} = \mathop \sum \limits_{j} p_{i,j} *r_{j,k}$$

where *Y*_*i,k*_ is the proportion of residents in community *i* with attitude *k* toward the vaccine (e.g., getting it as soon as possible), *p*_*i,j*_ is the proportion of residents in community *i* of race *j*, and *r*_*j,k*_ is the proportion of members of race *j* giving *k* as their response across the three surveys.

#### Cellphone generated mobility records

We used SafeGraph’s daily “Social Distancing” dataset to create the mobility network. The data are generated using a panel of GPS pings from anonymous mobile devices. Each device is attributed to an estimated home census block group (CBG) based on its most common nighttime location. It also tracks all stay points of these devices within other CBGs. The published data aggregate these pieces of information to generate a mobility matrix of the daily number of visits by the assumed residents of each CBG to each other CBG. Each CBG was nested in its ZIP code or municipality.

### Mobility-driven SIR model with the distribution of vaccination

#### Model

Our model was based on a traditional SIR (susceptible-infected-recovered) model that then incorporated two additional factors: mobility, to simulate the effect of contacts brought by the mobility between communities; and vaccination, to model the effect of the adoption of vaccination across communities. The full model consists of the following differential equations, which update daily:

$$\begin{aligned} \partial_{t} j_{n} & = \left( {\alpha_{n} j_{n} + \gamma \mathop \sum \nolimits_{m \ne n} \alpha_{n} w_{mn} j_{m} \frac{{N_{m} }}{{N_{n} }} + \gamma \mathop \sum \nolimits_{n \ne m} \alpha_{m} w_{nm} j_{m} } \right)s_{n} - \beta j_{n} \\ \partial_{t} s_{n} & = - \left( {\alpha_{n} j_{n} + \gamma \mathop \sum \nolimits_{m \ne n} \alpha_{n} w_{mn} j_{m} \frac{{N_{m} }}{{N_{n} }} + \gamma \mathop \sum \nolimits_{n \ne m} \alpha_{m} w_{nm} j_{m} + \mu g\left( {p_{n} } \right)} \right)s_{n} \\ \partial_{t} r_{n} & = \beta j_{n} \\ \partial_{t} p_{n} & = - g\left( {p_{n} } \right) + hu_{n} v_{n} \\ \partial_{t} u_{n} & = - hu_{n} v_{n} \\ \partial_{t} v_{n} & = g\left( {p_{n} } \right) \\ \end{aligned}$$

where *N*_*n*_, *j*_*n*_, *s*_*n*_, and *r*_*n*_ represent the total population size, number of infected cases, susceptible individuals, and recovered cases, respectively, for a community at a given timepoint.

The equations, which model simultaneous change over time in infected, susceptible, and recovered individuals, rely on four main components. α_*n*_ is the growth rate of infections in a community (i.e., the expected number of new cases from existing cases). It is based on a global α_0_ in combination with a sigmoid function that accounts for fluctuation effects between communities when *j*_*n*_ is less than a threshold ε i.e. $\alpha_{n} = \alpha_{0} \cdot \frac{{\left( {\frac{{j_{n} }}{\varepsilon }} \right)^{4} }}{{1 + \left( {\frac{{j_{n} }}{\varepsilon }} \right)^{4} }}$^48^. The second component pertains to mobility, including two operands: $r_{I} = \gamma \sum\nolimits_{m \ne n} {\alpha_{n} w_{mn} j_{m} \frac{{N_{m} }}{{N_{n} }}}$ calculates the possibility of an infected person in other communities (*m*) visiting the community and infecting susceptible in community *n*; and $r_{R} = \gamma \sum\nolimits_{n \ne m} {\alpha_{m} w_{nm} j_{m} }$ is the possibility of a susceptible person from the community visiting another community (*m*) and becoming infected. *w*_*mn*_ = *F*_*mn*_/*N*_*m*_ where *F*_*mn*_ indicates the total number of visits from community *m* to community *n*, as captured by the cell-phone generated mobility data. The average mobility rate gamma is defined as $\gamma = \frac{{\sum\nolimits_{m \in G} {F_{m} } }}{{\sum\nolimits_{m \in G} {N_{m} } }}$. The third component is the rate of recovery of infected individuals, represented by β, which is consistent across communities. All individuals who have been infected and recovered are permanently removed from the susceptible population.

The fourth component of the model is vaccination adoption, wherein *p*_*n*_ is the proportion of people who will definitely receive the vaccine, *u*_*n*_ is the proportion of people uncertain about getting the vaccine, and *v*_*n*_ is the proportion of people who have already been vaccinated. μ reflects the effectiveness of the vaccine and μ⋅*v*_*n*_ are treated as part of *r*_*n*_ as they have been removed from the susceptible population. *h* is the persuasion rate at which someone uncertain about getting the vaccine will be persuaded to do so, whose strength is contingent on the proportion of residents in the community who have already been vaccinated (i.e., *hu*_*n*_*v*_*n*_). We assume that *h* is consistent across communities. Only those who were uncertain about the vaccine could be persuaded, not those who stated they would never get the vaccine. Actual vaccination is represented by the function *g*(*p*_*n*_), of the form:

$$g\left( {p_{n} } \right) = \left\{ {\begin{array}{*{20}l} c \hfill & {p_{n} \ge c} \hfill \\ {p_{n} } \hfill & {p_{n} < c} \hfill \\ \end{array} } \right.$$

in which *c* is the maximum capacity of the vaccination rollout for a day.

#### Fixed parameters

Multiple parameters were established in advance of estimating the final model. α and β, the transmission and recovery rate, were estimated by running the simulation without vaccination on historical mobility and infection data for September 30th through December 22nd. Grid search identified a local optimum for α = 0.096 and β = 0.072, which translate to the more familiar *R*_0_ = α/β = 1.33. ε was calculated as $\varepsilon = \frac{M}{{\sum\nolimits_{m \in G} {N_{m} } }} = 3.826 \times 10^{ - 5}$, where *M* is the number of communities. μ, or the vaccine effectiveness, was set to 0.95, per the Pfizer and Moderna trial results. The rate of vaccine rollout was initially set to a 12-week (approx. 3-month) rollout period, meaning *c* = 1/(7*12) = 0.0119, or 1.19% of the population could be expect to be vaccinated daily.

In addition, we had to extend the simulation for three additional months for determining herd immunity (i.e., < 1 infection; the model permits fractions of infections), because only a third of communities had reached it by this definition at the end of the initial simulation. Two extreme scenarios are designed to impute the mobility data after Jan 12, 2021 to ensure that the real mobility data lies between the two imputed mobility datasets. We assume a cumulative 5 ± 0.5% increase or decrease for every 2 weeks to the real mobility data from Dec 29, 2020, to Jan 11, 2021.

For *h*, we assume that the rate at which individuals uncertain about taking the vaccine are persuaded to do so is dependent on the number of people in their community who have received the vaccine. The number of people a person knows in their neighborhood and the number of people they need to know who have been vaccinated to be persuaded both vary by individual. In terms of likelihood to be persuaded, we use an additional item from the Mass Inc-Museum of Science survey regarding when people would be likely to get vaccinated. We note two groups: those who would like to see a few people get the vaccine before they do, and those who would like to see many other people get it. We use the cross-tabs from this question with the initial vaccination intention question to distribute them within the sample (see Supplementary Online Materials), and differentiate between these functionally in the model based on how many members of their neighborhood need to be vaccinated for them to be persuaded. Numerous neighborhood surveys have found that people say they are friends with or personally know very few of those living in their neighborhood (e.g., 5 people on one’s street, less than 10 friends in the neighborhood^40^). Based on these numbers, we estimate that the average individual knows approximately 1% of the residents of his or her neighborhood well enough to know and relate to their vaccination experiences. We further estimate that if about 25% of these people (i.e., 0.25% of the population) were vaccinated, the average person would know at least “a few” people who had been vaccinated. Approximately 37% of people who were uncertain about vaccination said they would do so once “a few people they knew” were, the remainder when “many people” were. From this, we estimate that when 25% of the residents of a neighborhood are vaccinated, 18.5% of those who started out as uncertain will have been persuaded (that is, half of 37%, as only the average person would have enough exposure at that time). Based on the same results, we believe that the asymptote for persuasion is at 90%. We solved for these established points in a sigmoid function and found *h* = 0.026. All fixed parameters, their meaning, source, and value in the baseline model are reported in Table 2.

**Table 2:** Fixed values and sources for parameters in the baseline simulation model and robustness checks.

Parameter	Meaning	Value (robustness checks)	Source
α^a^	Transmission rate	0.096	Modeled on historical infections, mobility (9/30–12/22)
β^a^	Recovery rate	0.072	Modeled on historical infections
μ	Vaccine effectiveness	0.95 (0.75, 0.85)	Pfizer and Moderna trial results^26, 27^
*c*	Weekly rollout (% of population)	0.0119 (0.0089, 0.0056)	Based on 3-month (12-week) rollout
*h*	Persuasion rate	0.026 (0, 0.013, 0.039)	Sigmoid function based on neighborhood surveys about number of neighbors known^49^

^a^The more familiar *R*_0_ = α/β = 1.33.

#### Robustness tests

We ran iterations of the model to test for robustness, varying three elements: persuasion, vaccine efficacy, and timeline of vaccine roll-out. We tested persuasion as 50% stronger (*h* = 0.039) and 50% weaker (*h* = 0.013), as well as the absence of persuasion altogether (*h* = 0). We tested vaccine efficacy at the lower points of 85% and 75%. We tested the timelines for vaccination roll-out at four and 6 months (*c* = 0.0089 and 0.0056, respectively). We re-ran the simulation with all possible combinations, making for 36 sets of results (4 × 3 × 3). All parameters are reported in Table 2.

#### Methods statements

We confirm that all methods were carried out in accordance with relevant guidelines and regulations.

The survey “Living in Boston During COVID-19 Survey” conducted by the authors of this manuscript has IRB approval from Northeastern University and The University of Massachusetts Boston. Additionally, we confirm that informed consent was obtained from all subjects.

## Supplementary Information

Supplementary Information.

## Data Availability

The population descriptors from the U.S. Census’ American Community Survey’s 2014–2018 estimates were used from the processed and curated open database of BARI. According to the mobility data agreement, SafeGraph is providing free access to various datasets only to researchers, non-profits, and governments around the world which are working directly in the response to COVID-19, reason why this dataset is not open for reproducibility. The COVID-19 infection counts are openly available per week and day from the Massachusetts governmental website at town and county level. The COVID-19 infection counts for the City of Boston were processed from BPHC based on a shared data agreement, and we do not have the rights to publicly share the data. The vaccination information was derived from the Living in Boston survey, which was designed and implemented by the authors of this manuscript. The vaccination data is available upon request. Modeling code will be posted through GitHub upon publication of the paper.
