# Supplementary material for: Vaccination intentions generate racial disparities in the societal persistence of COVID-19
Source: Sci Rep. 2021 Oct 7;11:19906. doi: 10.1038/s41598-021-99248-2 (PMC8497595; doi:10.1038/s41598-021-99248-2)
Supplement: Supplementary file 1 — Supplementary Information. [file 41598_2021_99248_MOESM1_ESM.docx]

Supplementary Materials for

Title: Vaccination Intentions Generate Racial Disparities in the Societal Persistence of COVID-19

**This file includes:**

Figs. S1 to S3

Tables S1 to S7


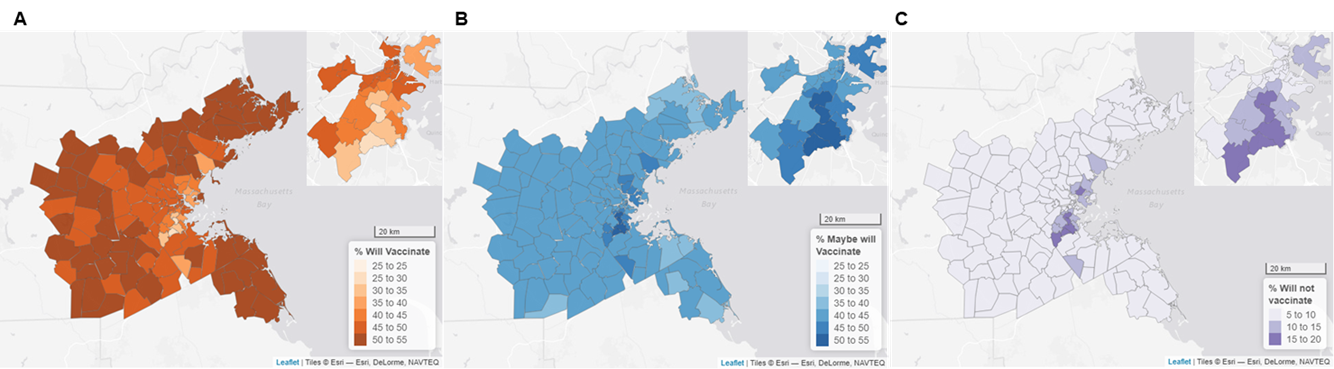


Fig. S1.

Representation of variations in vaccine intentions. Geographic differences in the proportion that (a) will definitely vaccinate, (b) maybe will vaccinate, and (c) definitely will not vaccinate. (Made in R Studio V.1.4. http://www.rstudio.com/)


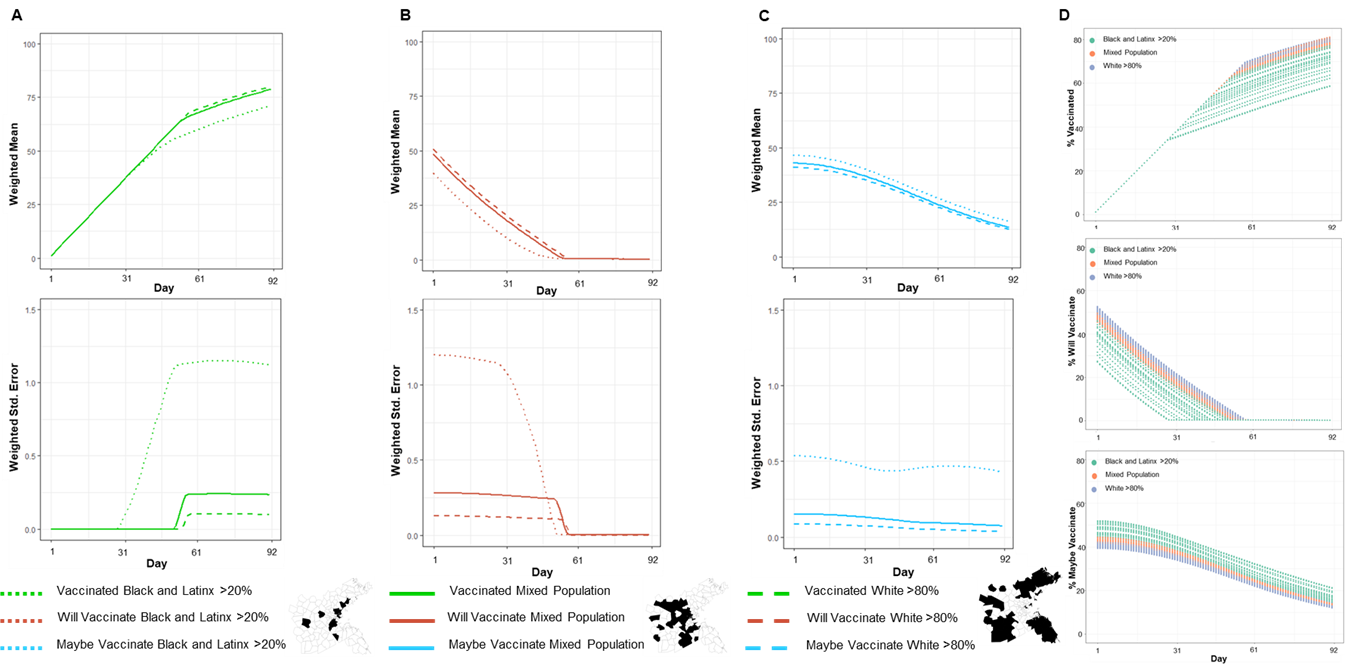


Fig. S2.

Disparities between communities of different ethnic composition across the simulation in (a) those vaccinated, (b) those will definitely vaccinate, and those who (c) maybe will vaccinate, including weighted mean across communities (top panel) and weighted standard error (bottom panel). (d) The same curves as in (a-c) for every individual community, categorized by racial composition.


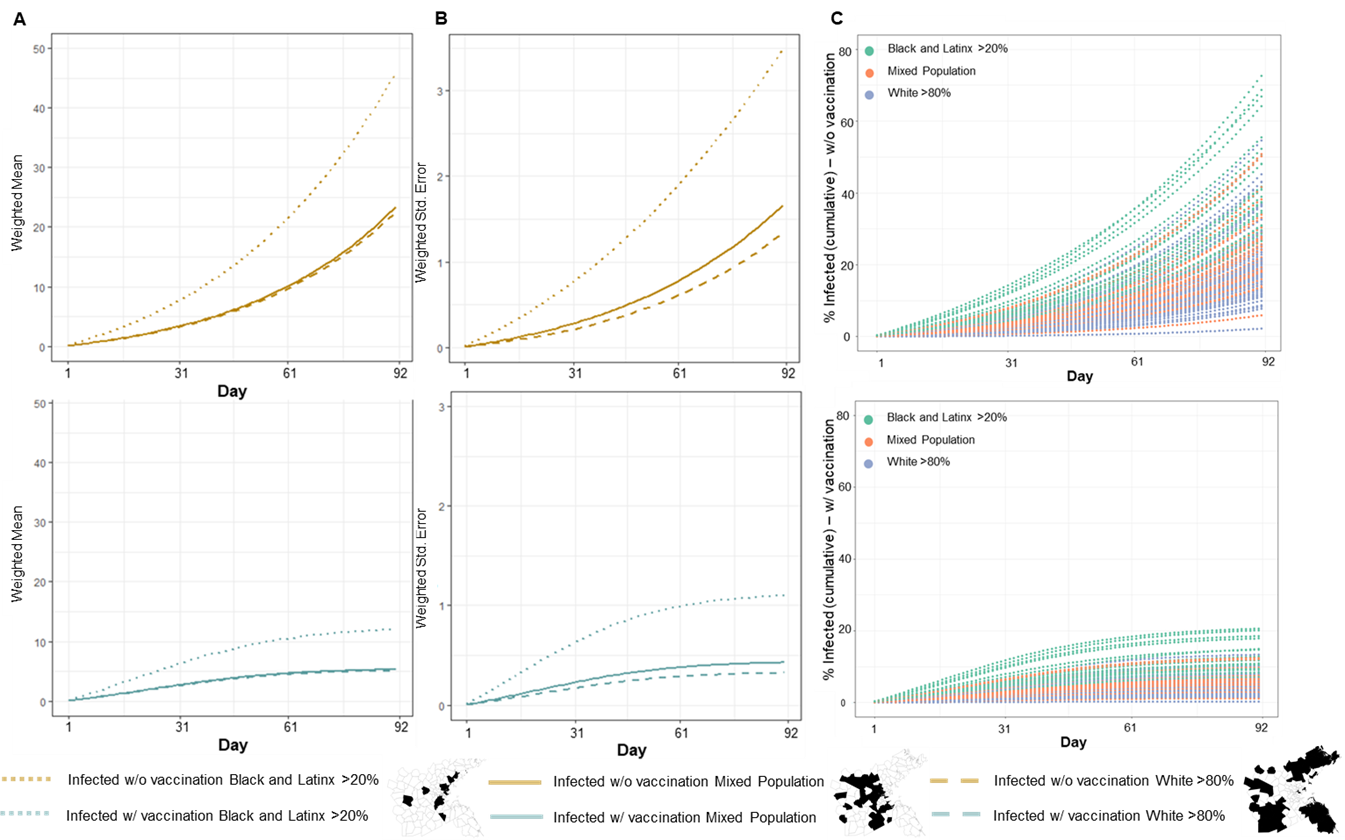


Fig. S3.

Community disparities shown for in the proportion of infected for the without vaccination (top panel) and with vaccination (bottom panel). (a) The weighted mean of infections by category of racial composition and (b) the weighted standard errors. (c) Cumulative values of proportion of infected residents, with one curve for every community, colored by category of racial composition.

Table S1.

Responses by race to the question “Will you get vaccinated?” in the Center for Survey Research-Boston Area Research Initiative survey.

|  | **Asian** | **Black** | **Latinx** | **White** | **Total** |
| --- | --- | --- | --- | --- | --- |
| *Definitely* | 44 (.49) | 21 (.25) | 24 (.34) | 350 (.56) | **439 (.51)** |
| *Probably* | 34 (.38) | 26 (.31) | 27 (.39) | 207 (.33) | **294 (.34)** |
| *Probably Not* | 9 (.10) | 21 (.25) | 11 (.16) | 50 (.08) | **91 (.11)** |
| *Definitely Not* | 2 (.02) | 16 (.19) | 8 (.11) | 14 (.02) | **40 (.05)** |
| **Total** | **89** | **84** | **70** | **621** | **864** |

Table S2.

Responses by race to the question “When an FDA-approved vaccine for COVID is made available, how likely will you be to take it?” in the Mass Inc.-Museum of Science survey.

|  | **Black** | **Latinx** | **White** | **Total** |
| --- | --- | --- | --- | --- |
| *Very likely* | 27 (.33) | 36 (.31) | 404 (.46) | **467 (.44)** |
| *Somewhat likely* | 23 (.28) | 28 (.24) | 246 (.28) | **297 (.28)** |
| *Not too likely* | 12 (.15) | 17 (.15) | 88 (.10) | **117 (.11)** |
| *Not at all likely* | 15 (.18) | 23 (.20) | 70 (.08) | **108 (.10)** |
| *Unsure* | 4 (.06) | 11 (.09) | 70 (.07) | **85 (.08)** |
| **Total** | **81** | **115** | **878** | **1073** |

Table S3.

Responses by race to the item “When a federally-approved COVID-19 vaccine is available to you, will you…” in the Suffolk-Boston Globe survey.

|  | **Asian** | **Black** | **Latinx** | **White** | **Total** |
| --- | --- | --- | --- | --- | --- |
| *Take it as soon as you can?* | 17 (.71) | 5 (.11) | 16 (.32) | 219 (.59) | **261 (.52)** |
| *Wait awhile until others have taken it?* | 7 (.29) | 24 (.55) | 21 (.42) | 107 (.29) | **164 (.33)** |
| *Not take the vaccine?* | 0 (.00) | 13 (.30) | 13 (.26) | 35 (.09) | **62 (.12)** |
| *Undecided* | 0 (.00) | 2 (.05) | 0 (.00) | 10 (.03) | **12 (.02)** |
| **Total** | **24** | **44** | **50** | **372** | **500** |

Table S4.

Cross-tabs between the items “When an FDA-approved vaccine for COVID is made available, how likely will you be to take it?” and “When an FDA-approved vaccine for COVID is made available, when do you think you will be most likely to take it?” in the Mass Inc.-Museum of Science survey.in the Suffolk-Boston Globe survey.

|  | *Very Likely* | *Somewhat likely* | *Not too likely* | *Not at all likely* |
| --- | --- | --- | --- | --- |
| *As soon as possible* | .72 | .13 | .03 | .01 |
| *After a few people I know have taken it* | .18 | .35 | .10 | .01 |
| *After many other people have taken it* | .08 | .45 | ,70 | .20 |
| *Never* | .00 | .00 | .05 | .59 |
| *Unsure* | .02 | .07 | .12 | .19 |

Table S5.

Parameter estimates (unstandardized betas, standard errors, and standardized betas) from regression equations meta-analyzing the impacts of different levels of persuasion, vaccine efficacy, and rollout rate on indicators of disparity from across simulations.

|  | **Effect of % Black** | | **Effect of % Latinx** | | **Effect of % Black (cont. inf.)** | | **Effect of % Latinx (cont. inf.)** | | **Diff. % Cmmtys w/ Imm.** | | **Diff. Avg. Date of Imm.** | |
| --- | --- | --- | --- | --- | --- | --- | --- | --- | --- | --- | --- | --- |
|  | **B (s.e.)** | **β** | **B (s.e.)** | **β** | **B (s.e.)** | **β** | **B (s.e.)** | **β** | **B (s.e.)** | **β** | **B (s.e.)** | **β** |
| *Persuasion* | -22.96*** (1.49) | -.90 | -25.99*** (4.33) | -.37 | -21.74*** (1.54) | -.84 | -16.93*** (1.61) | -.80 | -10.69** (3.44) | -.47 | -396.99*** (68.50) | -.46 |
| *Vaccine Efficacy* | -1.25*** (0.27) | -.27 | -5.01*** (0.77) | -.41 | -0.69* (0.27) | -.15 | -0.90** (0.29) | -.24 | -0.55 (0.61) | -.14 | -10.56 (9.43) | -.09 |
| *Rollout Rate*^a^ | 0.14  (0.09) | .10 | -3.00*** (0.25) | -.76 | 0.60*** (0.09) | .41 | 0.41*** (0.09) | .34 | -0.28 (0.20) | -.22 | 28.08*** (3.06) | .75 |

^*^p<0.05; **p<.01; ^***^p<0.001

^a^ – Measured as the percentage of the population that could be vaccinated each day under the assumed length of the rollout, meaning higher values reflect a faster rollout.

Table S6.

Comparisons of disparities across all simulations for herd immunity, including the percentage of predominantly White and high Black-Latinx communities that reach herd immunity and the average date of reaching herd immunity.

|  | **No Persuasion**  **(κ = 0)** | **Low Persuasion (κ = .013)** | **Medium Persuasion (κ = .026)** | **High Persuasion (κ = .039)** |
| --- | --- | --- | --- | --- |
| *Distribution Time = 3 months* | | | | |
| Vaccine Efficacy = 75% | 34% vs. 0%  139 days vs. NA | 100% vs. 18%  129 days vs. 178 days | 100% vs. 77%  115 days vs. 163 days | 100% vs. 100%  109 days vs. 155 days |
| Vaccine Efficacy = 85% | 80% vs. 0%  138 days vs. NA | 100% vs. 64% 111 days vs. 164 days | 100% vs. 100%  101 days vs. 149 days | 100% vs. 100%  97 days vs. 138 days |
| Vaccine Efficacy = 95% | 97% vs. 0%  121 days vs. NA | 100% vs. 86% 99 days vs. 153 days | 100% vs. 100%  91 days vs. 134 days | 100% vs. 100%  88 days vs. 125 days |
| *Distribution Time = 4 months* | | | | |
| Vaccine Efficacy = 75% | 28% vs. 0%  152 days vs. NA | 97% vs. 0%  142 days vs. NA | 100% vs. 59%  131 days vs. 171 days | 100% vs. 86%  127 days vs. 166 days |
| Vaccine Efficacy = 85% | 57% vs. 0%  141 days vs. NA | 100% vs. 50% 125 days vs. 171 days | 100% vs. 100%  116 days vs. 159 days | 100% vs. 100%  114 days vs. 150 days |
| Vaccine Efficacy = 95% | 95% vs. 0%  135 days vs. NA | 100% vs. 82% 112 days vs. 160 days | 100% vs. 100%  106 days vs. 144 days | 100% vs. 100%  104 days vs. 137 days |
| *Distribution Time = 6 months* | | | | |
| Vaccine Efficacy = 75% | 5% vs. 0%  169 days vs. NA | 45% vs. 0%  161 days vs. NA | 60% vs. 0%  162 days vs. NA | 65% vs. 0%  163 days vs. NA |
| Vaccine Efficacy = 85% | 28% vs. 0%  158 days vs. NA | 88% vs. 0% 157 days vs. NA | 95% vs. 18%  155 days vs. 179 days | 95% vs. 23%  155 days vs. 177 days |
| Vaccine Efficacy = 95% | 54% vs. 0%  150 days vs. NA | 98% vs. 32% 145 days vs. 178 days | 100% vs. 91%  144 days vs. 173 days | 100% vs. 95%  144 days vs. 171 days |

Table S7.

Comparisons of disparities across all simulations for infection rates, including the regression parameters for percentage Black and percentage Latinx, with and without controlling for infection rates under the no-vaccine scenario.

|  | **No Persuasion**  **(κ = 0)** | **Low Persuasion (κ = .013)** | **Medium Persuasion**  **(κ = .026)** | **High Persuasion (κ = .039)** |
| --- | --- | --- | --- | --- |
| *Distribution Time = 3 months* | | | | |
| Vaccine Efficacy = 75% | % Black = 1.44  % Latinx = 2.63  % Black (cont. inf.) = 1.25  % Latinx (cont. inf.) = 1.22 | % Black = 0.90  % Latinx = 1.70  % Black (cont. inf.) = 0.77  % Latinx (cont. inf.) = 0.76 | % Black = 0.54  % Latinx = 1.18  % Black (cont. inf.) = 0.45  % Latinx (cont. inf.) = 0.47 | % Black = 0.33  % Latinx = 0.91  % Black (cont. inf.) = 0.25  % Latinx (cont. inf.) = 0.30 |
| Vaccine Efficacy = 85% | % Black = 1.21  % Latinx = 2.00  % Black (cont. inf.) = 1.08  % Latinx (cont. inf.) = 1.01 | % Black = 0.72  % Latinx = 1.21  % Black (cont. inf.) = 0.63  % Latinx (cont. inf.) = 0.60 | % Black = 0.41  % Latinx = 0.80  % Black (cont. inf.) = 0.35  % Latinx (cont. inf.) = 0.35 | % Black = 0.24  % Latinx = 0.60  % Black (cont. inf.) = 0.19  % Latinx (cont. inf.) = 0.22 |
| Vaccine Efficacy = 95% | % Black = 1.01  % Latinx = 1.52  % Black (cont. inf.) = 0.92  % Latinx (cont. inf.) = 0.83 | % Black = 0.56  % Latinx = 0.87  % Black (cont. inf.) = 0.51  % Latinx (cont. inf.) = 0.46 | % Black = 0.30  % Latinx = 0.54  % Black (cont. inf.) = 0.26  % Latinx (cont. inf.) = 0.26 | % Black = 0.17  % Latinx = 0.39  % Black (cont. inf.) = 0.14  % Latinx (cont. inf.) = 0.16 |
| *Distribution Time = 4 months* | | | | |
| Vaccine Efficacy = 75% | % Black = 1.43  % Latinx = 2.99  % Black (cont. inf.) = 1.19  % Latinx (cont. inf.) = 1.18 | % Black = 0.90  % Latinx = 2.09  % Black (cont. inf.) = 0.71  % Latinx (cont. inf.) = 0.71 | % Black = 0.51  % Latinx = 1.60  % Black (cont. inf.) = 0.34  % Latinx (cont. inf.) = 0.38 | % Black = 0.28  % Latinx = 1.39  % Black (cont. inf.) = 0.12  % Latinx (cont. inf.) = 0.19 |
| Vaccine Efficacy = 85% | % Black = 1.23  % Latinx = 2.32  % Black (cont. inf.) = 1.05  % Latinx (cont. inf.) = 1.00 | % Black = 0.73  % Latinx = 1.55  % Black (cont. inf.) = 0.60  % Latinx (cont. inf.) = 0.58 | % Black = 0.39  % Latinx = 1.14  % Black (cont. inf.) = 0.28  % Latinx (cont. inf.) = 0.30 | % Black = 0.20  % Latinx = 0.97  % Black (cont. inf.) = 0.09  % Latinx (cont. inf.) = 0.15 |
| Vaccine Efficacy = 95% | % Black = 1.04  % Latinx = 1.79  % Black (cont. inf.) = 0.91  % Latinx (cont. inf.) = 0.84 | % Black = 0.59  % Latinx = 1.14  % Black (cont. inf.) = 0.50  % Latinx (cont. inf.) = 0.47 | % Black = 0.30  % Latinx = 0.81  % Black (cont. inf.) = 0.23  % Latinx (cont. inf.) = 0.23 | % Black = 0.15  % Latinx = 0.68  % Black (cont. inf.) = 0.07  % Latinx (cont. inf.) = 0.11 |
| *Distribution Time = 6 months* | | | | |
| Vaccine Efficacy = 75% | % Black = 1.20  % Latinx = 4.00  % Black (cont. inf.) = 0.75  % Latinx (cont. inf.) = 0.64 | % Black = 0.62  % Latinx = 3.61  % Black (cont. inf.) = 0.16  % Latinx (cont. inf.) = 0.23 | % Black = 0.43  % Latinx = 3.63  % Black (cont. inf.) = -0.03  % Latinx (cont. inf.) = 0.23 | % Black = 0.43  % Latinx = 3.63  % Black (cont. inf.) = -0.03  % Latinx (cont. inf.) = 0.23 |
| Vaccine Efficacy = 85% | % Black = 1.05  % Latinx = 3.25  % Black (cont. inf.) = 0.69  % Latinx (cont. inf.) = 0.58 | % Black = 0.51  % Latinx = 2.90  % Black (cont. inf.) = 0.15  % Latinx (cont. inf.) = 0.21 | % Black = 0.34  % Latinx = 2.91  % Black (cont. inf.) = -0.03  % Latinx (cont. inf.) = 0.21 | % Black = 0.34  % Latinx = 2.91  % Black (cont. inf.) = -0.03  % Latinx (cont. inf.) = 0.21 |
| Vaccine Efficacy = 95% | % Black = 0.92  % Latinx = 2.64  % Black (cont. inf.) = 0.63  % Latinx (cont. inf.) = 0.52 | % Black = 0.42  % Latinx = 2.32  % Black (cont. inf.) = 0.13  % Latinx (cont. inf.) = 0.18 | % Black = 0.27  % Latinx = 2.33  % Black (cont. inf.) = -0.02  % Latinx (cont. inf.) = 0.18 | % Black = 0.27  % Latinx = 2.33  % Black (cont. inf.) = -0.02  % Latinx (cont. inf.) = 0.18 |
